# Itaconate facilitates methane-induced Nrf2 pathway activation for mitigating liver ischemia and reperfusion injury

**DOI:** 10.1016/j.iliver.2025.100144

**Published:** 2025-02-13

**Authors:** Tianyi Zhang, Danfeng Fan, Kewei Qin, Hongtao Lu, Linwei Zhao, Kexin Liu, Pei Zhang, Qiang Sun, Zhouheng Ye

**Affiliations:** a Medical Innovation Research Department of PLA General Hospital, Beijing 100853, China; b Department of Hyperbaric Medicine, The Sixth Medical Center of PLA General Hospital, Beijing 100048, China; c College of Otolaryngology Head and Neck Surgery, The Sixth Medical Center of PLA General Hospital, Beijing 100048, China; d Department of Naval Medicine, Naval Medical University, Shanghai 200433, China; e School of Medicine, South China University of Technology, Guangzhou 518055, China; f Department of Radiation Oncology, The Fifth Medical Center of PLA General Hospital, Beijing 100071, China; g Department of Special Operations Medicine, The Sixth Medical Center of PLA General Hospital, Beijing 100048, China

**Keywords:** Methane, Itaconate, Nrf2, Ischemia, Reperfusion, Acod1, Liver

## Abstract

***Background and aims*:**

Methane has shown protective effects against ischemia and reperfusion injury (IRI) in the liver, but the mechanism underlying these beneficial effects is unclear. To investigate the hypothesis that itaconate facilitates in methane-induced Nrf2 pathway activation to mitigate liver IRI.

***Methods*:**

An oxygen and glucose derivation (OGD) model using RAW 264.7 cells and a liver IRI model in mice were established. Methane's beneficial effects were assessed through hematoxylin and eosin (HE) staining, Suzuki's score, serum alanine transferase level, superoxide dismutase (SOD) level, malondialdehyde (MDA) level, and cell viability. The relative expression levels of Nrf2, its downstream molecules and some inflammatory factors were detected via western blotting. Itaconate levels were analyzed using liquid chromatography. RAW 264.7 cells were transfected with short hairpin RNA targeting mouse aconitate decarboxylase 1 (Acod1) mRNA for itaconate downregulation.

***Results*:**

Methane significantly alleviated liver IRI, as shown by the significant reduction in Suzuki's scores and alanine transferase (ALT) levels in vivo. Methane treatment significantly increased MTT and SOD levels and decreased MDA levels in the OGD injury model in vitro. Methane also increased the total and nuclear Nrf2 expression levels, activated downstream molecules including heme oxygenase-1 (HO-1), NQO1 and affected the production of inflammatory cytokines such as IL-10, IL-1β, and IL-12. Itaconate levels were significantly elevated after methane treatment compared with the OGD injury group. The protective effects of methane were abolished after itaconate downregulation through *Acod1* knockdown.

***Conclusions*:**

Methane alleviates liver IRI through itaconate/Nrf2 pathway activation, with itaconate being critical for methane's beneficial effects.

## Graphical abstract

Methane increases mitochondrial itaconate production to activate the Nrf2 pathway.

**Figure:**
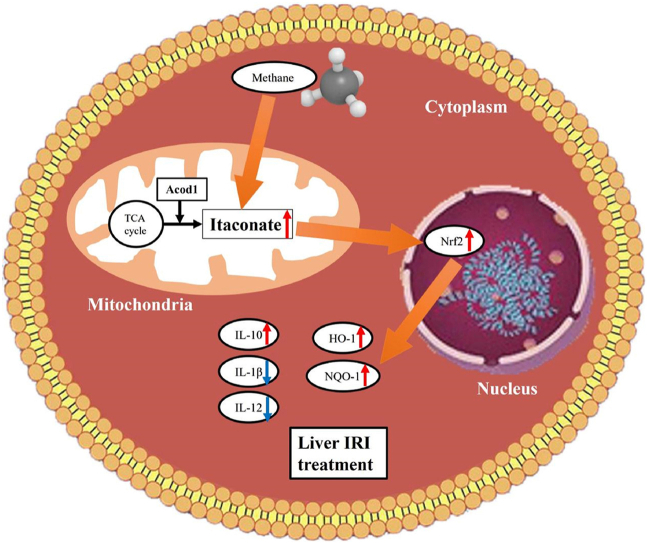

## 1 Introduction

Liver ischemia and reperfusion injury (IRI) is a clinical condition occurring after liver transplantation, ischemic shock, or liver trauma.^1^ Currently, there are no practical and effective drugs for treating an IR injury. Our previous work has shown that methane gas holds promise as a therapeutic agent for alleviating liver IRI through an anti-oxidative pathway without causing adverse effects.^2^ However, the key mechanism underlying the beneficial effects of methane remains unclear, hindering its translation of methane from research to clinical practice.

Transcription factor NF E2-related factor 2 (Nrf2) is a major sensor of oxidative stress during hepatic IR injury.^3^ After liver IR injury, Nrf2 activation triggers the transcription of various molecular pathways, such as heme oxygenase-1 (HO-1), NAD(P)H quinone oxidoreductase 1 (NQO1), glucose-6-phosphate dehydrogenase (G6PDH), neuronal nitric oxide synthase (nNOS), glutamate cysteine ligase catalytic subunit (GCLC), glutamate cysteine ligase modifier subunit (GCLM), and glutathione S-transferases (GSTs) which are essential for maintaining cellular homeostasis.^4^, ^5^, ^6^ While Nrf2 protein has certain functions in the nucleus, it is stabilized in the cytoplasm by Keap1 (Kelch-like ECH-associated protein 1, Keap1) under normal conditions. Methane has been shown to activate Nrf2 in other ischemia-reperfusion injury models^7^; however, the exact mechanism by which methane influences Nrf2 activation remains unknown.

Itaconate, a metabolite in the tricarboxylic acid cycle, occurs at a low physiological concentration in mitochondria. It is synthesized from cis-aconitic acid through the catalysis of cis-aconitate decarboxylase, an enzyme encoded by *immune-responsive gene 1* (*IRG1*) Itaconate is produced in larger amounts during perfusion injury, toxic injury, and other oxidative-related injuries. Itaconate is transported from the mitochondria to the cytoplasm, where it binds to Keap1, thereby activating the Nrf2 (NF-E2-related factor 2, Nrf2) protein.^8^ Artificially synthesized 4-octyl itaconate can exogenously activate Nrf2^9^; thus, itaconate is an upstream molecule of Nrf2.^10^ Our preliminary experiments suggest that methane may react with itaconate, enhancing its production and transport efficiency, thereby playing a protective role against oxidative injury through the activation of Nrf2.

In this paper, we established a reperfusion injury model to investigate the relationship between methane and itaconate. Using both in vivo and in vitro injury models, we examined Nrf2 activation and increase in itaconate levels. Additionally, we used a knockdown technique in vitro to downregulate itaconate, allowing us to observe how methane affects itaconate.

## 2 Materials and methods

### 2.1 Animals

C57BL/6N mice were purchased from Cyagen Bioscience Inc. (Guangzhou, China). Male mice aged 6–8 weeks and weighing 24–28 g were used in the experiments. A total of 23 mice were housed and maintained under a 12 h light/dark cycle with free access to water and food. The study protocol was approved by the Animals Ethics Committee of the Chinese PLA General Hospital and Institutional Animal Care and Use Committee (IAGUC) of Cyagen Biomodels Company (ACU21-A074).

### 2.2 Liver IR injury model

The classical model of warm liver IRI was applied in the experiment, as described previously.^2^ Briefly, the arterial and portal venous blood supply to the left and middle lobes of the liver was obstructed using an atraumatic vascular clamp. Anesthesia was induced with 2.5% isoflurane and maintained throughout the procedure via the intraperitoneal administration of chloral hydrate (0.1 mL/10 g). The ischemic phase lasted for 60 min, followed by a reperfusion injury phase of 6 h. Mice assigned to the sham group underwent the same procedure as mice in the other groups, except that their vessels were not occluded. The mice were killed immediately 6 h after reperfusion. Immediately upon the onset of reperfusion, methane saline (10 mL/kg) was injected. The methane saline used in this study was prepared using a natural dissolution method. The concentration of methane obtained through the natural dissolution and high-pressure methods was determined (Supplementary Fig. 1).

### 2.3 HE staining and Suzuki's score

Liver tissues were embedded with paraffin and sliced into 5 mm sections. The sections were stained with hematoxylin and eosin (HE). A light microscope (Olympus, Tokyo, Japan) was used to capture HE images. Suzuki's score was used to assess injury, as described in a previous procedure.^11^ The injury assessment included grading for congestion, vacuolization, and necrosis, with each parameter scored from 0 to 4. The score for each slide was determined by the addition of all these scores. Suzuki's scores for each slide ranged from 0 to 12.

### 2.4 Serum alanine transferase measurements

Serum samples were collected after 12 h of precipitation and centrifuged for 10 min at 4000 rpm and 4 °C. Serum alanine transferase (ALT) levels were analyzed using an autoanalyzer (Hitachi 7600Y20, Japan), and results were presented in U/L.

### 2.5 Cell culture

Raw267.4 mouse macrophage cells (purchased from PUHE Biotechnology, CTCC-ACL-0131) were cultured in a culture medium (DMEM/F-12 culture medium, L340KJ, from Shanghai Basal Media Technologies Co.) supplemented with 10% calf serum, 3.15 g/L D-glucose, 2.5 mM L-glutamine and 55 mg/L sodium pyruvate at 37 °C in a 75%N_2_–20%O_2_–5%CO_2_ incubator. For experiments, cells were transferred to 96-well plates at a concentration of 5 × 10^4^ cells/mL.

### 2.6 Cell grouping

Cells were categorized based on two types of grouping methods. The first grouping method involved the classification of cells into three groups: (1) Sham group: Cells were not subjected to oxygen and glucose deprivation (OGD) injury. (2) OGD group: Cells underwent OGD injury. (3) OGD + CH_4_ group: Cells underwent OGD injury and were treated with methane. The second grouping method involved the classification of cells into five groups. (1) Raw264.7 + scramble: Raw264.7 cells were transfected with control siRNA; (2) Raw264.7 + scramble + OGD: Raw264.7 cells were transfected with control siRNA and subjected to OGD injury; (3) Raw264.7 + shIrg1 + OGD: Raw264.7 cells were transfected with IRG1 siRNA and subjected to OGD injury; (4) Raw264.7 + scramble + OGD + methane: Raw264.7 cells were transfected with control siRNA, subjected to OGD injury, and treated with methane; (5) Raw264.7 + shIrg1 + OGD + methane: Raw264.7 cells were transfected with IRG1siRNA, subjected to OGD injury, and treated with methane.

### 2.7 OGD injury model

The OGD injury model involved subjecting cells to oxygen and glucose deprivation. First, cells were cultured in a glucose-free medium and placed in a 95%N_2_–5%CO_2_ incubator for 6 h. Next, the cells were returned to their culture medium and cultured under normal air conditions in a 75%N_2_–20%O_2_–5%CO_2_ incubator for 48 h. Cells in the control group were cultured under normal air conditions during the entire procedure. Cells in the methane group were cultured in a 71.5%N_2_–20%O_2_–5%CO_2_-3.5%CH_4_ incubator during the 48 h.

### 2.8 IRG1 knockdown in RAW 264.7 cell lines

*IRG1* knockdown was performed in RAW 264.7 cell lines by transfecting the cells with short hairpin RNA (shRNA) targeting mouse aconitate decarboxylase 1(ACOD1, Gene ID 16365) mRNA. Three different shRNAs were designed to increase *IRG1* knockdown efficiency. Raw 264.7 cell lines transfected with scramble RNA served as controls. The efficiency of IRG1 knockdown was verified using qPCR and drug screening (Supplementary Fig. 2).

### 2.9 Western blotting

Cells and tissues were homogenized in RIPA lysis buffer with protease and phosphatase inhibitors (Beyotime Chemical Co., China). The bicinchoninic acid assay (BCA) (Beyotime Chemical Co., China) was used to determine protein concentrations in cell and tissue samples, and each sample was balanced based on the mean protein concentration. Equal protein amounts, i.e., 25 μg per lane, were loaded onto 15% SDS-PAGE gels, and proteins were transferred to a nitrocellulose membrane (Beyotime Chemical Co., China). The membrane was blocked with 5% nonfat milk in TBST overnight at 4 °C and incubated with primary antibodies for 2 h at room temperature. An ECL chemiluminescence system (Bestbio, Shanghai, China) was applied for band detection. Image J software was used to determine relative protein expression levels. Antibodies against HO-1 (1:1000), NQO-1 (1:10,000), IL-12 (1:1000), IL-10 (1:1000), Nrf2 (1:1000), and GAPDH (1:5000) were used in this study.

### 2.10 SOD and MDA measurement

Liver tissues were homogenized in 10 mL of physiological saline, followed by centrifugation at 3000 rpm. Cells were homogenized with 500 μL physiological saline after centrifugation at 2000 rpm. SOD (Superoxide dismutase) concentrations were determined using the thiobarbituric acid method (Jiancheng Institute of Biotechnology, Nanjing, China). MDA (Malondialdehyde) concentrations were calculated using a nucleotide oxidation inhibition method (Beyotime Chemical Co., shanghai, China). OD values were determined using a spectrophotometer (AQ8100, Thermo Scientific, Waltham, Massachusetts, USA) at respective absorbance values.

### 2.11 MTT detection

Cells were transferred to a 96-well plate by adding 100 μL of the cell solution into each well (4 × 10^4^ cells/mL). After adding 10 μL of MTT(3-(4,5-dimethylthiazol-2-yl)-2,5-diphenyltetrazolium bromide) solution to each well, the wells were cultured in an incubator for 4 h. The solution in the wells was then discarded and replaced with 50 μL DMSO. After agitation of the solution using a shaker at room temperature, OD values were detected using a spectrophotometer (AQ8100, Thermo Scientific, USA) at 492 nm.

### 2.12 Itaconate analysis using a liquid chromatograph

Cells were lysed in 300 μL methanol, homogenized for 5 min, and sonicated for 10 min at 4 °C. The supernatant was collected after centrifugation for 10 min at 12,000 rpm. Itaconate levels were analyzed using a liquid chromatograph (Q Exactive, Thermo Fisher Scientific, USA). A standard curve was constructed using itaconate-methanol mixtures at 2000 ng/mL, 500 ng/mL, 200 ng/mL, 50 ng/mL, and 20 ng/mL. Chromatograms were processed using Xcalibur 4.0 software (Thermo Fisher Scientific, USA).

### 2.13 Statistical analysis

Statistical analysis was conducted with SPSS software 22.0 (SPSS Inc., Chicago, USA). Measurement data were presented as mean ± SD values. Enumeration data were presented as median values. Data from three or more groups were analyzed using a one-way analysis of variance (ANOVA) followed by Tukey's test for normally distributed data. Non-normally distributed data from multiple groups were analyzed with one-way ANOVA followed by Dunnett's T3 test. Enumeration data for multiple groups were analyzed using the nonparametric test followed by the Nemenyi Test. A *p* < 0.05 was considered statistically significant.

## 3 Results

### 3.1 Methane could treat liver IRI injury in vivo

**Fig. 1:**
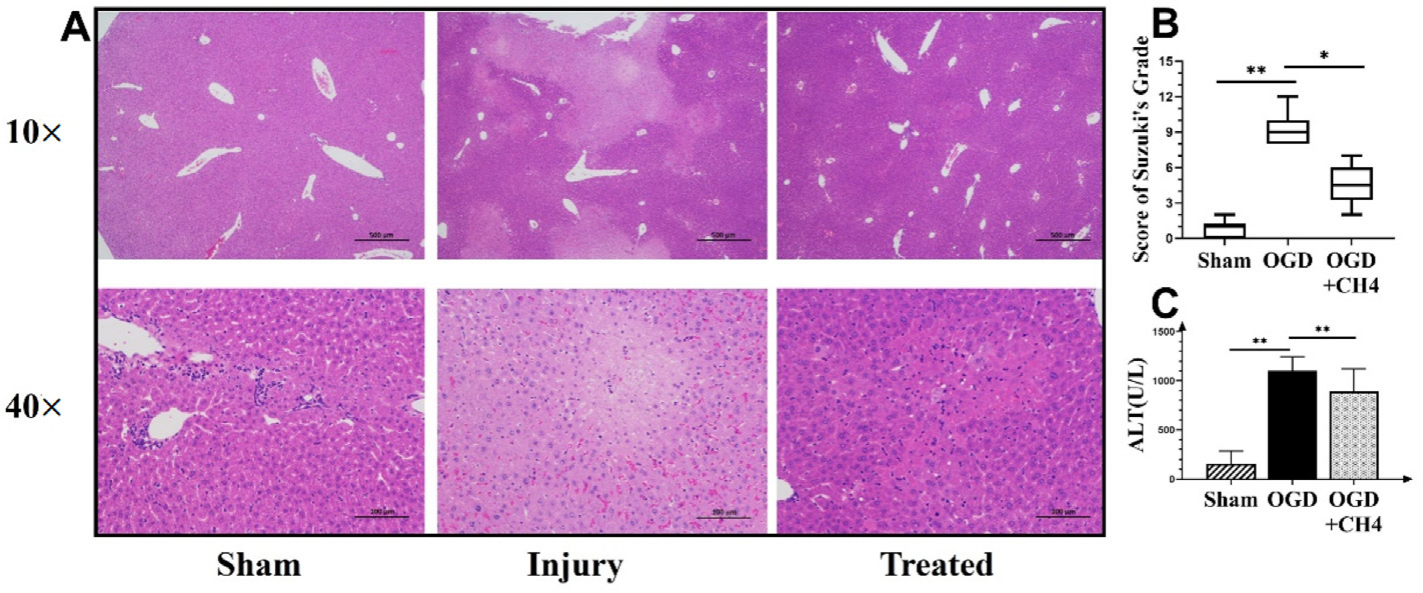
Methane could treat liver IRI injury in vivo. A: HE staining of liver tissue. The upper row shows a representative image of liver sections at 10 × magnification (scale bar: 500 μm), while the bottom row shows the corresponding image at 40 × magnification (scale bar: 100 μm). B: Suzuki's score after HE staining to identify liver injury in sham, liver IR injury, and methane-treated groups. Data are presented as median ± SD values, with a sample size of 6–9 per group. C: Serum ALT levels in the sham, liver IR injury, and methane-treated groups. Data are presented as mean ± SD values, with a sample size of 7–9. ∗*p* < 0.05, ∗∗*p* < 0.01. Abbreviations: ALT: Serum alanine transferase. HE: hematoxylin and eosin.

To verify the protective effects of methane on IRI injury, Suzuki's scores for liver tissue HE staining and serum ALT levels were used as indicators. Post-IR injury, liver sections showed extensive congestion, vacuolization, and necrosis compared to the sham group, as shown in Fig. 1A. Methane treatment alleviated liver injury in all three fields. Fig. 1B presents a box diagram of Suzuki's scores used to measure and compare the damage within groups. While the sham group had a low score (1.0 ± 0.7), the injury group showed a significantly higher (9.0 ± 1.3) score (*p* = 0.0008). The score in the methane-treated group was significantly decreased to 4.5 ± 1.6 compared to the injury group (*p* = 0.03). The ALT level in the injury group increased significantly to 1099.1 ± 146.7 U/L compared to the sham group (*p* < 0.01) (Fig. 1C). After methane treatment, the ALT level was decreased significantly to 890.6 ± 228.0 U/L compared to the injury group (*p* = 0.023).

### 3.2 Methane could treat cell OGD injury in vitro

**Fig. 2:**
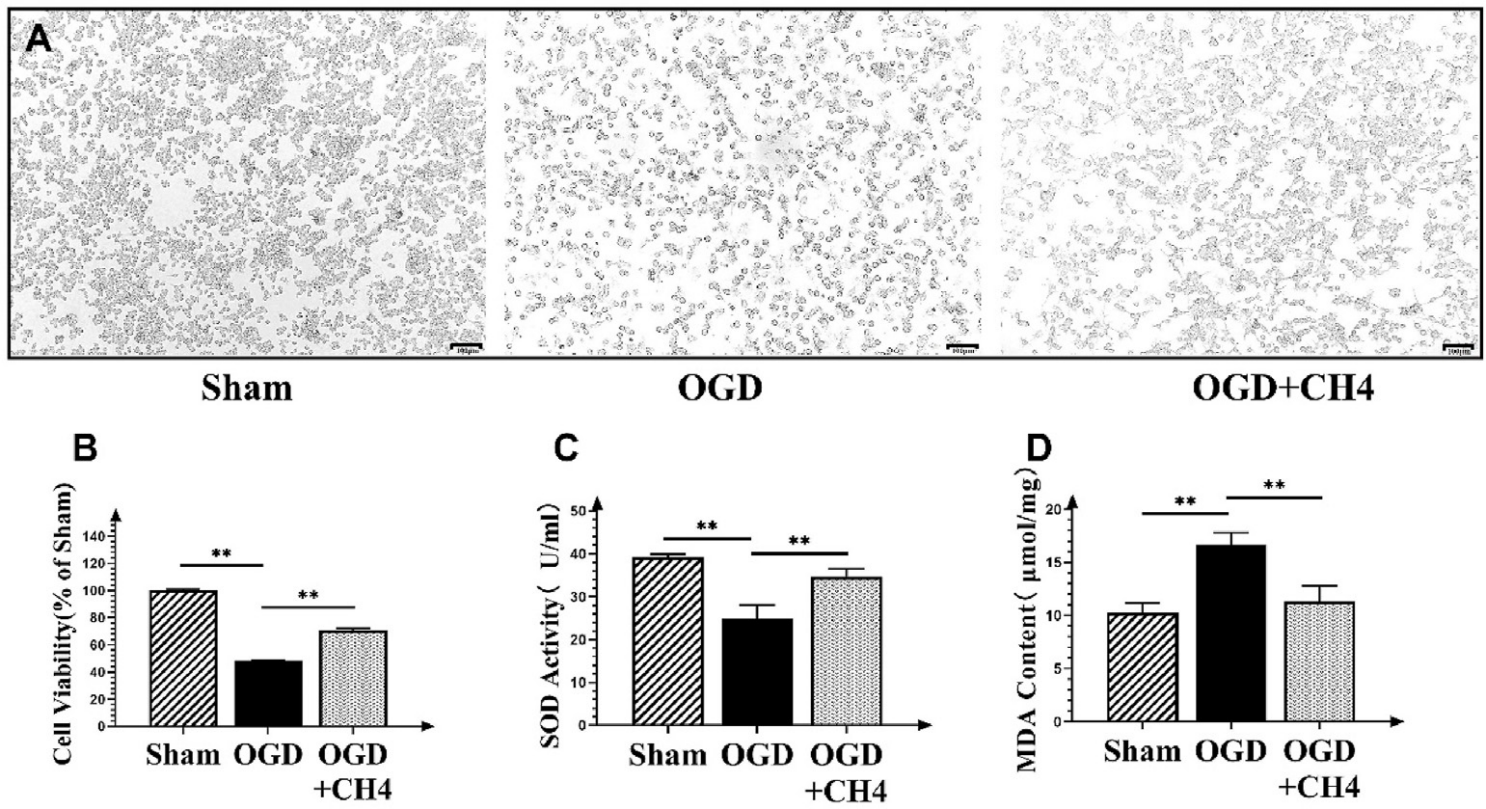
Methane could treat cell OGD injury in vitro. A: An image of cells from the sham, OGD, and OGD + CH_4_ groups at 40 × magnification (Scale bar:100 μm). B: MTT assay results for the sham, OGD, and OGD + CH_4_ groups. Data are presented as mean ± SD values, with a sample size of 3. ∗*p* < 0.05, ∗∗*p* < 0.01. C: SOD levels in the sham, OGD, and OGD + CH_4_ groups. Data are presented as mean ± SD values, with a sample size of 3. ∗*p* < 0.05, ∗∗*p* < 0.01. D: MDA levels in the sham, OGD, and OGD + CH_4_ groups. Data are presented as mean ± SD values, with a sample size of 3. ∗*p* < 0.05, ∗∗*p* < 0.01. Abbreviations: MTT: 3-(4,5-dimethylthiazol-2-yl)-2,5-diphenyltetrazolium bromide; OGD: oxygen and glucose deprivation; CH_4_: Methane; SOD: Superoxide dismutase; MDA: Malondialdehyde.

In vitro studies were conducted to verify the protective effects of methane, using cell staining, MTT assays, and measurements of SOD and MDA levels as indicators. Cells were spindle-shaped and clustered in the sham group, and ballon-shaped and scattered in the OGD group. After methane treatment, a greater number of cells were maintained in the spindle-shaped and clustered state. The MTT assay showed that cell viability dropped significantly to 48.48% compared to the sham group (*p* < 0.01). The cell viability of the OGD + CH_4_ group was increased significantly to 68.40% (*p* < 0.01) (Fig. 2B). In Fig. 2C, SOD levels in the OGD group decreased significantly (24.80 ± 3.29) compared to the sham group (39.24 ± 0.66, *p* < 0.01). Methane treatment significantly increased SOD levels to 34.68 ± 1.83 compared to the OGD group (*p* = 0.004). As shown in Fig. 2D, MDA levels increased significantly in the OGD group (16.67 ± 1.15) compared to the sham group (10.25 ± 0.92, *p* = 0.002). Methane treatment significantly reduced MDA levels to 11.31 ± 1.49 compared to the OGD group (*p* = 0.004). These results showed that methane effectively alleviated OGD-induced injury, increased cell viability, reduced MDA production, and increased SOD levels. The data from both in vivo and in vitro studies demonstrated that methane could mitigate liver IRI injury.

### 3.3 Involvement of Nrf2 in the protective effects of methane

**Fig. 3:**
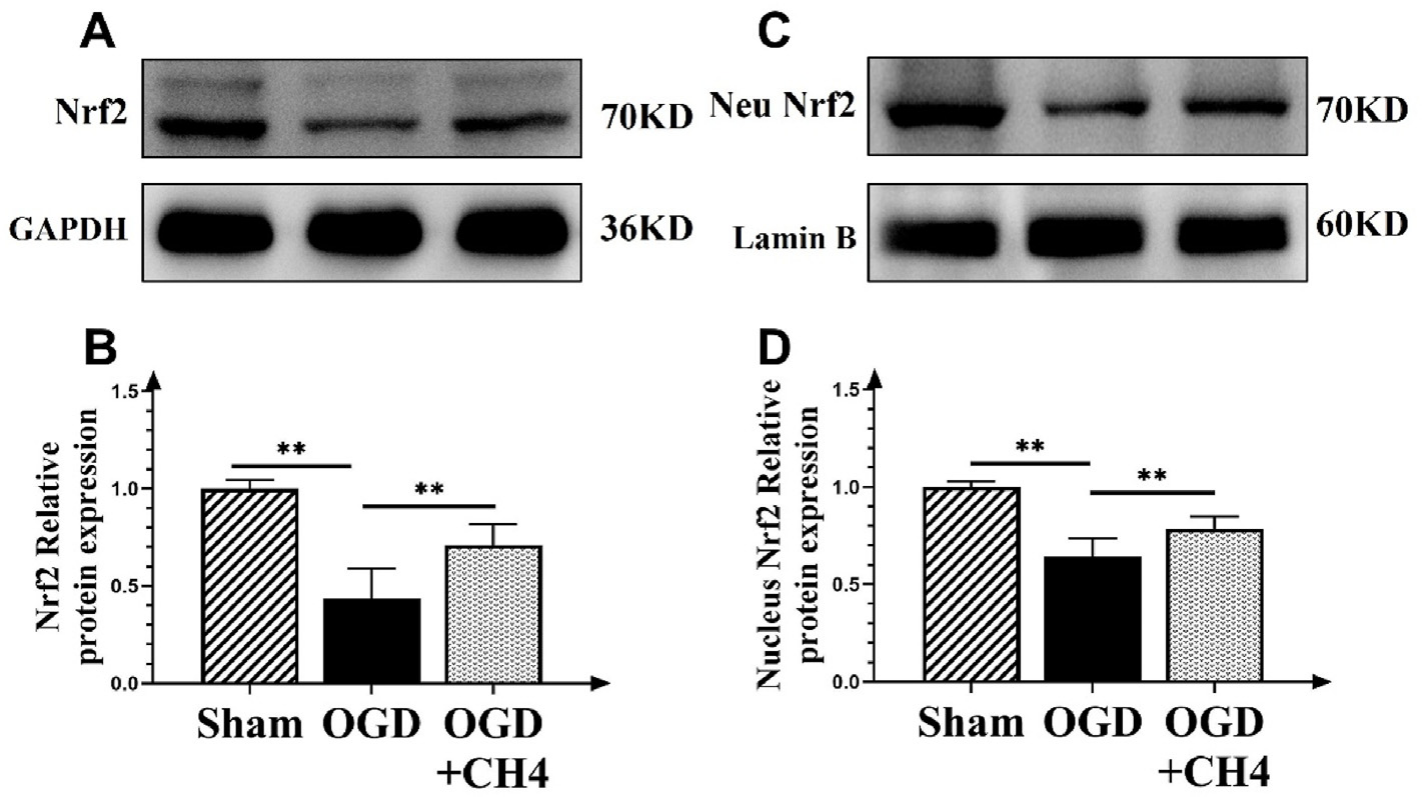
The involvement of Nrf2 in the protective effects of methane. A: Representative Western blot of total relative Nrf2 expression in the sham, OGD, and OGD + CH_4_ groups. B: Relative total Nrf2 protein expression in the sham, OGD, and OGD + CH_4_ groups. Data are presented as mean ± SD values, with a sample size of 9. ∗*p* < 0.05, ∗∗*p* < 0.01. C: Representative Western blot of relative nuclear Nrf2 expression in the sham, OGD, and OGD + CH_4_ groups. D: Relative nuclear Nrf2 protein expression in the sham, OGD, and OGD + CH_4_ groups. Data are presented as mean ± SD values, with a sample size of 9. ∗*p* < 0.05, ∗∗*p* < 0.01. Abbreviations: OGD: oxygen and glucose deprivation; CH_4_: Methane; GAPDH: glyceraldehyde-3-phosphate dehydrogenase.

The relative expression levels of total cellular Nrf2 protein and nuclear Nrf2 proteins are shown in Fig. 3. The band corresponding to total Nrf2 is shown in Fig. 3A. The OGD group showed a thinner band compared to the sham group, while the OGD + CH_4_ group exhibited a heavier band compared to the OGD group. The total relative protein expression is shown in Fig. 3B. The relative expression in the OGD group decreased to 0.43 ± 0.16, which is significantly lower compared to the sham group (*p* < 0.01). The relative total Nrf2 expression in the OGD + CH_4_ group increased to 0.71 ± 0.12, which is significantly higher compared to the OGD group (*p* = 0.002). A similar trend was observed for nuclear Nrf2, both in the representative band of nuclear Nrf2 and the statistical results of relative expression levels (OGD: 0.64 ± 0.10 vs OGD + CH_4_: 0.79 ± 0.06, *p* = 0.006).

### 3.4 Involvement of the Nrf2 pathway in the protective effects of methane

**Fig. 4:**
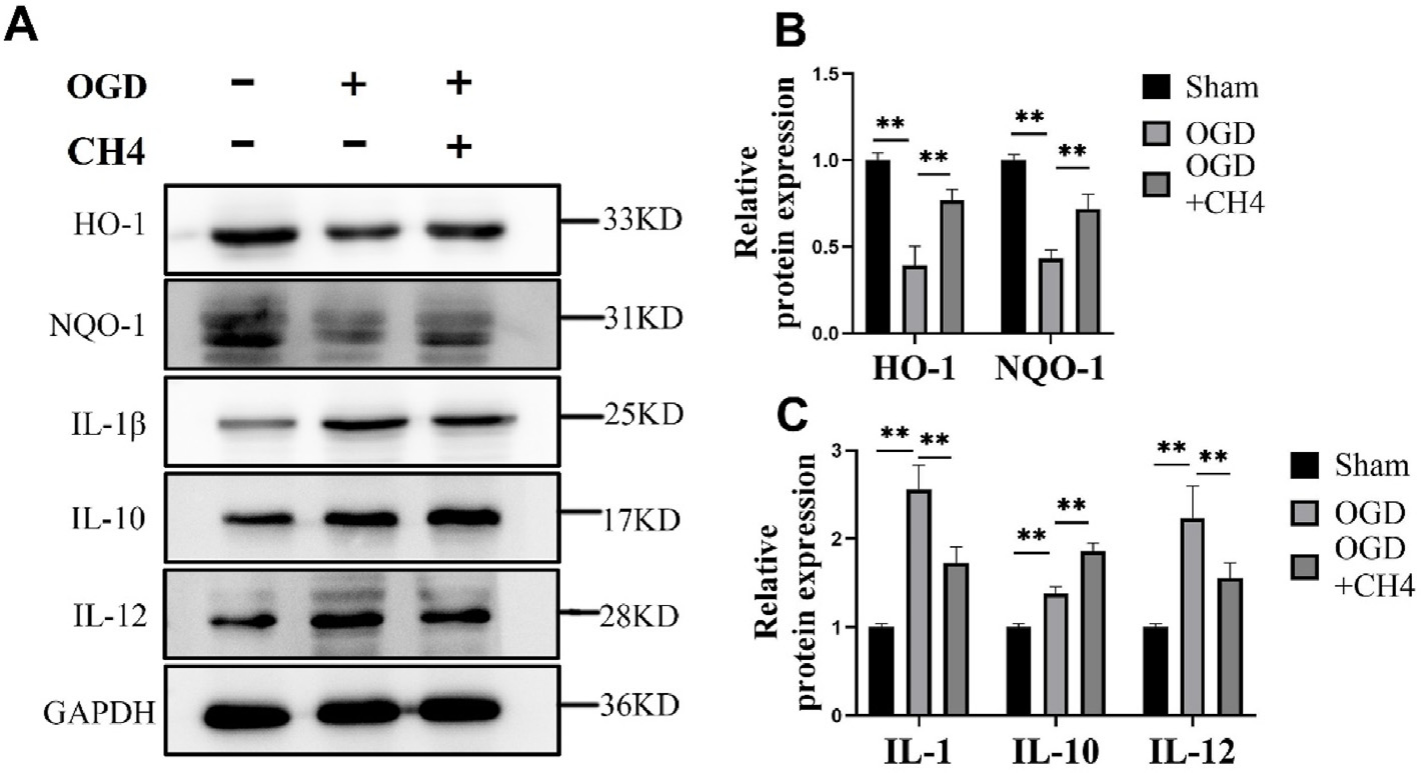
Involvement of the Nrf2 pathway in the protective effects of methane. A: Representative Western blot of HO-1, NQO-1, IL-1β, IL-10, and IL-12 expression in the sham, OGD, and OGD + CH_4_ groups. B and C: Relative expression of HO-1, NQO-1, IL-1β, IL-10, and IL-12 proteins in the sham, OGD, and OGD + CH_4_ groups. Data are presented as mean ± SD values, with a sample size of 9. ∗*p* < 0.05, ∗∗*p* < 0.01. Abbreviations: OGD: oxygen and glucose deprivation; CH_4_: Methane; HO-1: Heme oxygenase-1; NQO-1: NAD(P)H Quinone Dehydrogenase 1; IL-1β: Interleukin 1β; IL-10: Interleukin 10; IL-12: Interleukin 12; GAPDH: glyceraldehyde-3-phosphate dehydrogenase.

To investigate the involvement of the Nrf2 pathway and its effects on the inflammatory factors, HO-1, NQO-1, IL-1β, IL-10, and IL12 levels were assessed using Western blot analysis (Fig. 4). The expression of HO-1 was decreased to 0.39 ± 0.11 in the OGD group, which is significantly lower compared to the sham group (*p* < 0.01). After methane treatment, the HO-1 level significantly increased to 0.77 ± 0.06 in the OGD + CH_4_ group compared to the OGD group (*p* < 0.01). A similar trend was observed for the relative protein expression of NQO-1 (OGD: 0.43 ± 0.05 vs OGD + CH_4_: 0.72 ± 0.09, *p* < 0.01). A similar tendency was also observed for IL-10 (OGD: 1.38 ± 0.07 vs OGD + CH_4_: 1.86 ± 0.09, *p* < 0.01). The relative protein level of IL-1β was significantly higher (2.56 ± 0.27) in the OGD group than in the sham group (*p* < 0.01). Methane treatment significantly reduced IL-1β levels in the OGD + CH_4_ group to 1.73 ± 0.18 compared to the OGD group (*p* < 0.01). A similar pattern was observed with IL-12 protein levels (OGD: 2.24 ± 0.36 vs OGD + CH_4_: 1.56 ± 0.17, *p* = 0.001).

### 3.5 Involvement of itaconate in the protective effects of methane

**Fig. 5:**
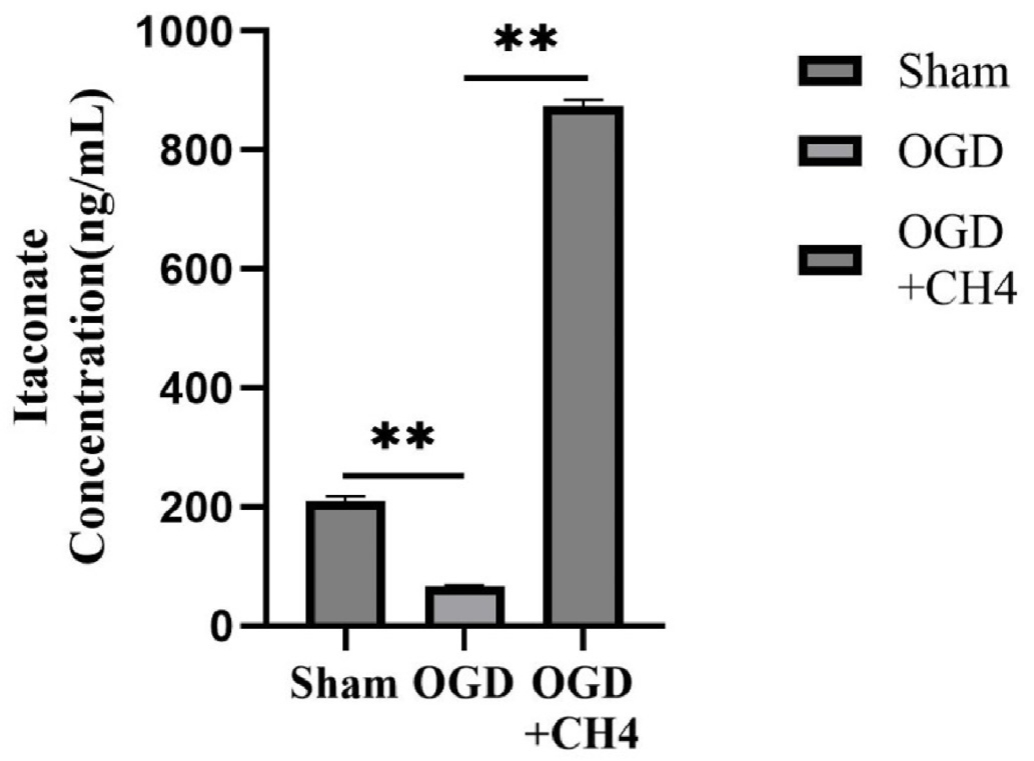
Itaconate plays a role in the protective effects of methane. The itaconate levels in the sham, OGD, and OGD + CH_4_ groups were determined using the liquid chromatograph method. Data are presented as mean ± SD values, with a sample size of 3. ∗*p* < 0.05, ∗∗*p* < 0.01. Abbreviations: OGD: oxygen and glucose deprivation; CH_4_: Methane.

To further explore the role of itaconate, its levels were measured in RAW264.7 cells. As shown in Fig. 5, the itaconate level dropped significantly to 65.85 ± 2.68 in the OGD group compared to the sham group (210.35 ± 7.89, *p* < 0.01). After methane treatment, itaconate levels were markedly elevated (874.75 ± 9.82) compared to the OGD group (*p* < 0.01). These results showed that methane affects not only the Nrf2 pathway but also upstream metabolite itaconate.

### 3.6 Itaconate knockdown diminished the protective effects of methane

**Fig. 6:**
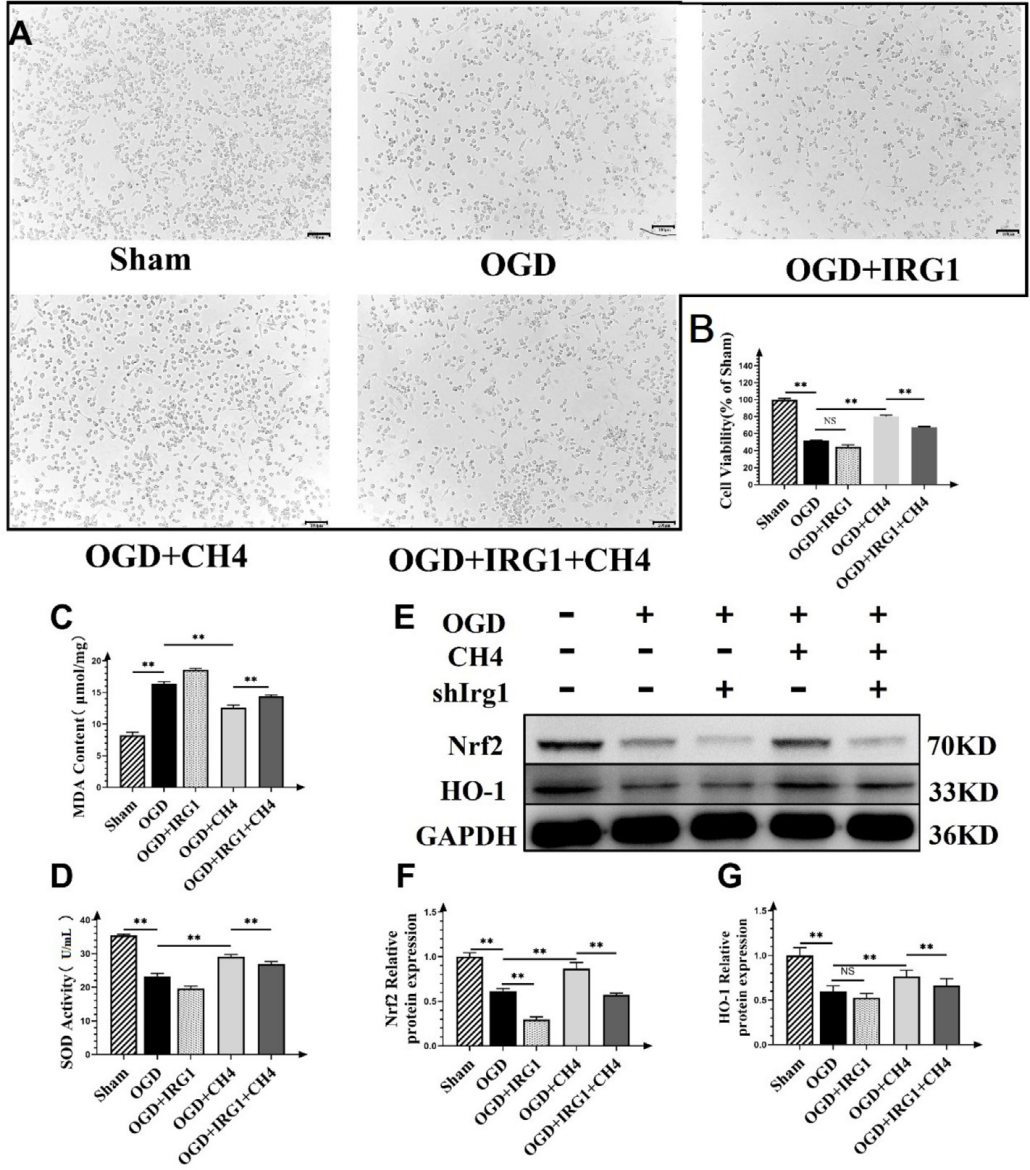
Itaconate knockdown diminished the protective effects of methane. A: An image of cells at 40 × magnification (scale bar: 100 μm) in the sham, OGD, OGD + IRG1, OGD + CH_4_, and OGD + IRG1 + CH_4_ groups. B: MTT assay results for the five groups. C: MDA levels in the five groups. Data are presented as mean ± SD values, with a sample size of 3. ∗*p* < 0.05, ∗∗*p* < 0.01. D: SOD levels in five groups. Data are presented as mean ± SD values, with a sample size of 3. ∗*p* < 0.05, ∗∗*p* < 0.01. E: Western blot of Nrf2 and HO-1 relative expression levels in the five groups. F and G: Relative expression of Nrf2 and HO-1 proteins in the five groups. Data are presented as mean ± SD values, with a sample size of 9. ∗*p* < 0.05, ∗∗*p* < 0.01. Abbreviations: OGD: oxygen and glucose deprivation; CH_4_: Methane; IRG1: immune-responsive gene 1; NS: not significant; SOD: Superoxide dismutase; MDA: Malondialdehyde; shIrg1: Irg1 knockdown using short hairpin RNA; HO-1: Heme oxygenase-1; GAPDH: glyceraldehyde-3-phosphate dehydrogenase; NS: Not Significant.

To confirm the critical role of itaconate in the protective effects associated with methane treatment, *Acod1* was knocked down using a lentivirus transfection method. Fig. 6A showed that cells in the sham group grew in clusters and exhibited a shuttled shape. The number of cells declined substantially in the OGD group, and cells appeared scatted and bubble-shaped. After IRG1 knockdown, the cell number decreased further and the cell morphology became abnormal. After methane treatment, the cell counts increased, with a proportion of cells maintaining a shuttle shape. These protective effects were diminished by IRG1 knockdown in the OGD + IRG1 + CH_4_ group. To quantify these observations, the cell viability was measured using the MTT assay (Fig. 6B). The cell viability of the sham group was set at 100%. In the OGD injury group, the cell viability dropped significantly to 51.95% ± 0.31%. After IRG1 knockdown, the cell viability decreased to 44.59% ± 2.23%, which was significantly lower compared to the sham group (*p* < 0.01) but no significant difference was observed compared to the OGD group (*p* = 0.103). After methane treatment, the cell viability increased to 80.44% ± 1.46%, which is significantly higher compared to the OGD group (*p* = 0.002). This effect was partially reversed (dropped to 67.78% ± 0.74%) after IRG1 knockdown in the OGD + IRG1 + CH_4_ group compared to the OGD + CH_4_ group. Fig. 6C shows that the MDA level in the OGD group was increased to 16.35 ± 0.36 μmoL/mg, which was significantly higher compared to the sham group (8.22 ± 0.50, *p* < 0.01). Methane treatment reduced the MDA level to 12.62 ± 0.39 μmoL/mg, which was significantly lower compared to the OGD group (*p* < 0.01). After IRG1 knockdown, the MDA level increased to 14.41 ± 0.19 μmoL/mg compared to the OGD + CH_4_ group (*p* < 0.01). SOD activity in the sham group was 35.45 ± 0.33 U/mL (Fig. 6D). It decreased significantly to 23.32 ± 0.83 U/mL after OGD injury (*p* < 0.01). After methane treatment, the SOD level increased significantly to 29.08 ± 0.72 U/mL (*p* < 0.01). After IRG1 knockdown, the SOD level decreased again to 26.92 ± 0.76 U/mL. It was significantly lower than that of the OGD + CH_4_ group (*p* = 0.023). Western blot analysis of Nrf2 pathway proteins showed a lighter band for Nrf2 protein than that observed in the scramble group (Fig. 6E). The band became darker after methane treatment but lightened again after IRG1 knockdown in the OGD + CH_4_+ IRG1 group, a trend also observed with the HO-1 protein. Fig. 6J and K shows the relative expression levels of Nrf2 and HO-1. In the OGD group, the Nrf2 protein level was 0.62 ± 0.03, which was significantly lower than that in the sham group (*p* < 0.01). Methane treatment increased the relative Nrf2 protein expression to 0.87 ± 0.07 which was significantly higher than that in the OGD group (*p* < 0.01). After IRG1 knockdown, Nrf2 protein expression decreased to 0.67 ± 0.02, which was significantly lower compared to the OGD + CH_4_ group (*p* < 0.01). The relative expression of HO-1 was lower in the OGD group (0.60 ± 0.06) compared to the sham group (*p* < 0.01). After methane treatment, HO-1 expression increased to 0.76 ± 0.07, which was significantly higher than that in the OGD group (*p* < 0.01). After IRG1 knockdown, the Nrf2 protein expression was reduced to 0.66 ± 0.07 in the methane treatment group, which was significantly lower compared to the OGD + CH_4_ group (*p* = 0.031).

## 4 Discussion

Our previous study demonstrated that methane could treat liver IRI.^2^ However, the mechanism underlying these beneficial effects remained unclear. We proposed that methane could protect against hepatic IR injury through the itaconate/Nrf2 pathway. First, methane alleviated liver IR injury, as shown in both an in vivo hepatic IR injury model and an in vitro OGD injury model. Second, the itaconate/Nrf2 pathway was involved with the protective effects of methane on liver IR injury, with methane treatment activating Nrf2 pathway. Third, itaconate was identified as a key target in the modulation of the itaconate/Nrf2 pathway by methane, essential for protecting against liver IRI. The protective effects of methane on cell viability, SOD, MDA levels, and the expression of Nrf2 and HO-1 were abolished after itaconate knockdown. Thus, the activation of the itaconate/Nrf2 pathway may be central to the protective effects of methane on liver IRI injury.

The Nrf2 pathway plays a critical role in the protective effects of methane on IR diseases and liver injury. By activating Nrf2, methane could treat lung ischemia/reperfusion,^7^ spinal ischemia/reperfusion,^12^ and myocardial ischemia/reperfusion.^13^ Treatment with Nrf2 siRNA significantly hampered the antioxidant and protective effects of methane in vitro.^13^ Methane alleviates acetaminophen-induced liver injury through the Nrf2 pathway and regulates the Nrf2/HO-1/NQO1 signaling pathway both in vivo and in vitro during injury.^14^ These findings demonstrate the critical role of Nrf2 in the protective effects of methane against ischemia-reperfusion injury and liver injury, making Nrf2 activation a focus of interest for researchers across various fields.

We focused on macrophages rather than hepatocytes because macrophages are the primary cells responsible for orchestrating the inflammatory response during ischemia-reperfusion injury (IRI). Macrophages, particularly Kupffer cells and infiltrating monocytes, are key players in the innate immune response, rapidly producing pro-inflammatory cytokines and chemokines upon activation. This inflammatory cascade is central to the pathogenesis of liver IRI, making macrophages an essential target for understanding the underlying mechanisms.^15^ Additionally, recent studies have highlighted the role of macrophages in itaconate production,^16^ an immunomodulatory metabolite with anti-inflammatory properties that is crucial for modulating the inflammatory response. This metabolic pathway is specifically associated with macrophage activation and is a key area of interest in IRI research.^17^

Methane could enhance the protective effects of itaconate on liver IR injury by increasing the permeability of the mitochondrial membrane, to allow the translocation of itaconate. Itaconate was majorly accumulated in the mitochondria,^18^ but during hypoxic stress, mitochondrial homeostasis was disrupted.^19^ Consequently, some of the itaconate went through the mitochondrial membrane into the cytoplasm.^20^ In the cytoplasm, the concentration of itaconate becomes elevated, and it binds to the Keap1 protein, triggering Nrf2 activation.^16^ However, due to its low lipid solubility, itaconate has limited membrane permeability. In contrast, 2-methyl itaconate, with its high lipid solubility, crosses the mitochondrial membrane more easily.^21^^,^^22^ Methane might facilitate this process by donating a methyl group to itaconate, increasing its lipid solubility and potentially forming 2-methyl itaconate. This hypothesis warrants further investigation, particularly with regard to the conditions under which methane interacts with itaconate. Future work on mitochondrial membrane permeability will build upon these findings and contribute to a more comprehensive mechanistic model.

Regarding the clinical application of methane, several key issues need to be addressed: (1) Administration Methods: Methane can be administered via inhalation or through intraperitoneal injection, depending on the experimental design. In clinical applications, methane could be administrated orally twice a day. Also, inhalation may be more suitable for clinical applications due to its non-invasive nature. (2) Timing: The optimal timing of methane administration should be determined through preclinical studies. Early intervention may be crucial for maximizing its protective effects, especially in acute injury models. (3) Safety and Efficacy: Future studies should focus on evaluating the safety and efficacy of methane in larger animal models and, eventually, in clinical trials. The use of controlled dosing and monitoring of potential side effects will be essential for translating these findings to clinical practice.

## 5 Conclusion

Our study demonstrated that methane mitigates liver IRI injury through the itaconate/Nrf2 pathway, with itaconate probably serving as a key factor mediating the protective effects of methane. Our work supports the potential clinical application of methane in treating liver IR injury-related diseases in the future.

## 6 Limitations

Several limitations are associated with our study. First, in vivo research was limited. The itaconate concentration was not tested in the liver IR injury model, and IRG1 inhibition was not replicated in the animal model. Second, our sample size was small. Third, the theory of methane reacting with itaconate in the mitochondria was not validated via chemical experiments. Fourth, the mechanistic insights into how methane promotes itaconate production are still limited, and further studies are needed to fully elucidate the underlying mechanisms within the cells. Fifth, in our study, we demonstrated the necessity of itaconate through Acod1 knockdown experiments. However, future work should explore the sufficiency of itaconate by overexpressing ACOD1 or using itaconate derivatives.

## CRediT authorship contribution statement

**Tianyi Zhang:** Writing – original draft, Software, Project administration, Methodology, Formal analysis. **Danfeng Fan:** Writing – original draft, Project administration, Methodology, Investigation, Formal analysis. **Kewei Qin:** Project administration, Funding acquisition. **Hongtao Lu:** Project administration. **Linwei Zhao:** Project administration. **Kexin Liu:** Project administration. **Pei Zhang:** Formal analysis, Data curation. **Qiang Sun:** Writing – review & editing, Supervision, Conceptualization, Formal analysis. **Zhouheng Ye:** Writing – review & editing, Supervision, Resources, Funding acquisition, Conceptualization.

## Informed consent

Not applicable.

## Any other ethical approval

Not applicable.

## Data availability statement

The data that support the conclusions of this study can be made available by the corresponding author upon reasonable request.

## Declaration of Generative AI and AI-assisted technologies in the writing process

Not applicable.

## Animal treatment

The study protocol was approved by the Animals Ethics Committee of the Chinese PLA General Hospital and Institutional Animal Care and Use Committee (IAGUC) of Cyagen Biomodels Company (ACU21-A074).

## Organ donation

Not applicable.

## Generative AI

Not applicable.

## Funding

This research was funded by 10.13039/501100001809National Natural Science Foundation of China (82000587, 82202382) and the 10.13039/501100004826Beijing Municipal Natural Science Foundation (7242135).

## Declaration of competing interest

The authors and funders declare no conflict of interest.
