# Supplementary material for: Itaconate facilitates methane-induced Nrf2 pathway activation for mitigating liver ischemia and reperfusion injury
Source: ILIVER. 2025 Feb 13;4(1):100144. doi: 10.1016/j.iliver.2025.100144 (PMC12212682; doi:10.1016/j.iliver.2025.100144)
Supplement: Multimedia component 1 [file mmc1.docx]

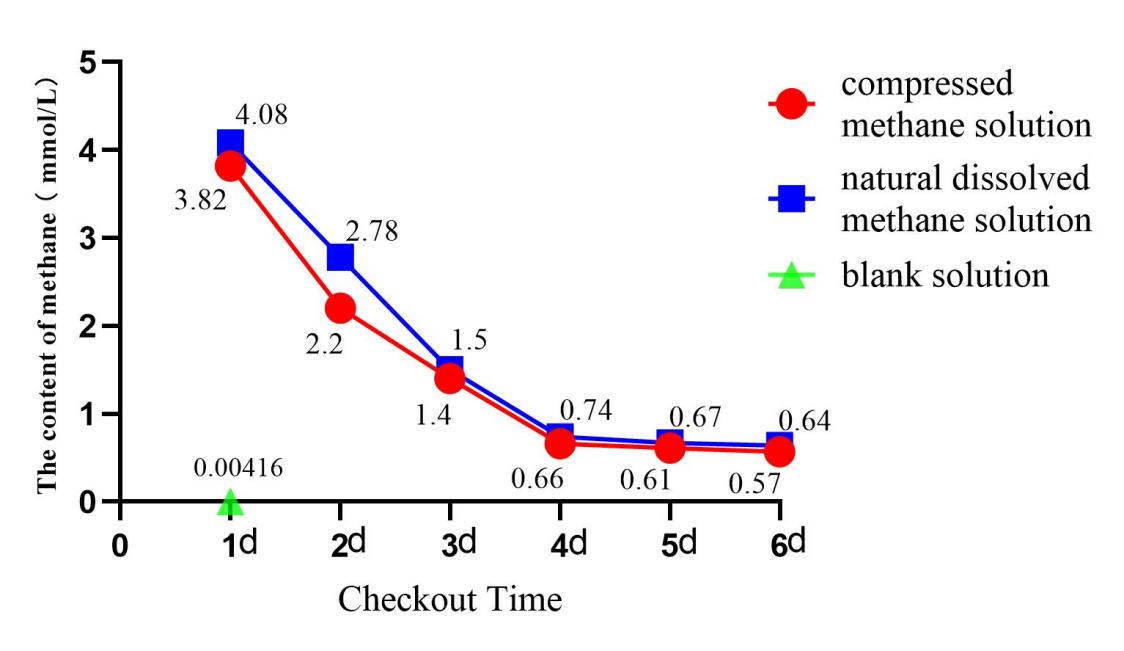


Supplementary Fig. 1. Determining the concentration of produced methane solution via two independent methods: Circle marks indicate the use of the compressed methane method. Square marks represent the use of the natural dissolved methane method, which is the method used in this study. Triangle marks represent the controls, with no methane in the solution.


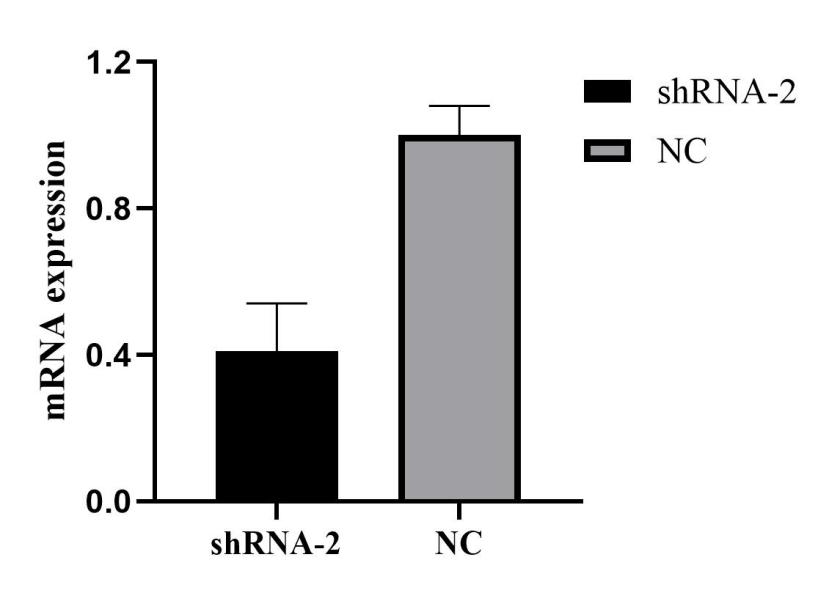


Supplementary Fig. 2. The mRNA levels of the shRNA-2 and control groups.

Abbreviations: NC: negative control.
